# Tumor location and patient characteristics of colon and rectal adenocarcinomas in relation to survival and TNM classes

**DOI:** 10.1186/1471-2407-10-688

**Published:** 2010-12-21

**Authors:** Kari Hemminki, Irene Santi, Marianne Weires, Hauke Thomsen, Jan Sundquist, Justo Lorenzo Bermejo

**Affiliations:** 1 Division of Molecular Genetic Epidemiology, German Cancer Research Center (DKFZ), Im Neuenheimer Feld 580, D-69120 Heidelberg, Germany; 2 Center for Primary Health Care Research, Lund University, Malmö, Sweden; 3 Stanford Prevention Research Center, Stanford University School of Medicine, Palo Alto, California, USA; 4 Institute of Medical Biometry and Informatics (IMBI), University Hospital Heidelberg, Germany

## Abstract

**Background:**

Old age at diagnosis is associated with poor survival in colorectal cancer (CRC) for unknown reasons. Recent data show that colonoscopy is efficient in preventing left-sided cancers only. We examine the association of Tumor Node Metastasis (TNM) classes with diagnostic age and patient characteristics.

**Methods:**

The Swedish Family-Cancer Database has data on TNM classes on 6,105 CRC adenocarcinoma patients. Ordinal logistic regression analysis was performed to model tumor characteristics according to age at diagnosis, tumor localization, gender, socioeconomic status, medical region and family history. The results were compared to results from survival analysis.

**Results:**

The only parameters systematically associated with TNM classes were age and tumor localization. Young age at diagnosis was a risk factor for aggressive CRC, according to stage, N and M with odds ratios (ORs) ranging from 1.80 to 1.93 for diagnosis before age 50 years compared to diagnosis at 80+ years. All tumor characteristics, particularly T, were worse for colon compared to rectal tumors. Right-sided tumors showed worse characteristics for all classifiers but M. The survival analysis on patients diagnosed since 2000 showed a hazard ratio of 0.55 for diagnosis before age 50 years compared to diagnosis at over 80 years and a modestly better prognosis for left-sided compared to right-sided tumors.

**Conclusions:**

The results showed systematically more aggressive tumors in young compared to old patients. The poorer survival of old patients in colon cancer was not related to the available tumor characteristics. However, these partially agreed with the limited colonoscopic success with right-sided tumors.

## Background

Mortality in colorectal cancer (CRC) has declined in the developed countries because the incidence has no longer increased and because the survival has improved [1-3]. Adenocarcinoma is the most common histology of colon and rectal cancers. The 5-year survival for colon adenocarcinoma reached about 60% in Sweden towards the end of the 1990s being a few percentage points better for women compared to men [4]; for rectal adenocarcinoma the male survival was about 55% compared to over 60% for women. The reasons for the improvements in survival have been suggested to be earlier diagnosis and better health status and care [4,5]. However, the favorable development has not benefitted equally all patients. The survival in elderly patients is poorer than that of the young patients [6]. Nevertheless, the differences in survival and treatment response tend to be small in clinical trials on fit elderly patients [7,8]. The age differences are larger for colon than rectal cancer in most but not all studies [9]. In colon cancer, the risk of dying is about twice as high as in those diagnosed at age over 75 years compared to those diagnosed before age 60 years [10,11]. The reasons for the differential survival have been unclear and factors such as late seeking of care, co-morbidities, less aggressive treatment and discontinued treatment have been invoked [6,9,12,13]. If delayed diagnosis were the reason for the poor survival, tumor characteristics would show more advanced stages in older patients. Results on tumor characteristics of colorectal tumors have been ambiguous but the recent data on colon cancer tend to point to the opposite: younger patients present more aggressive and advanced tumors [11,14-16]. High education level has been shown to correlate with better survival in colon cancer and somewhat better survival in rectal cancer [17]. Family history of particularly colon cancer appears to be associated with better survival, which has also been observed for hereditary nonpolyposis colorectal cancer (HNPCC) patients [18-20].

The Swedish Cancer Registry has recently started to record tumor characteristics based on the TNM classification. We use these data, accumulated over four years between 2004 and 2008, to assess the distribution of the characteristics of the patients and their proximal and distal colon cancers and rectal cancer according to stage and TNM classes in a total of 6,105 CRC adenocarcinoma patients. We carried out also a survival study by tumor localization on patients diagnosed since 2000. The TNM findings are discussed in terms of age-dependent survival and recent evidence that colonoscopy is effective in preventing left-sided tumors only [21,22].

## Methods

The population-based Swedish Family-Cancer Database was created by linking the Multigeneration Register at Statistics Sweden to the Swedish Cancer Registry [23-25]. The Multigeneration Register includes individuals born in Sweden after 1931 and their biological parents, providing registered information of the Swedish families through the past century [24,25]. The Swedish Cancer Registry is based on compulsory reports about patients provided by pathologists and cytologists, who report every cancer diagnosis on surgically removed tissue, biopsies, cytological specimens, bone marrow aspirates and autopsies [23]. Both the diagnostic accuracy and coverage are believed to approach 100%; in recent years over 5000 CRCs were annually diagnosed and between 100-150 cases were additionally reported in death certificates but not included in the Cancer Registry [23]. The Database comprises more than 12 million individuals and over 1 million first primary cancers [24,25]. Data on patients with cancer were retrieved from the Swedish Cancer Registry from 1958 to 2008. International Classification of Disease (ICD-7) codes 153.0 to 153.3 and 154.0 were used for CRC and 120,125 cases were identified, 93% of which were adenocarcinomas, according to the pathological anatomic diagnosis code 096. Based on the codes, the anatomic location of colon was classified as right-sided sections (proximal, codes 153.0 and 153.1) and left-sided sections (distal, codes 153.2 and 153.3). The splenic flexure was the dividing line of the left and the right locations.

Tumor depth of invasion, nodal status and presence of metastatic disease were available since 2002 according to the TNM system introduced by the American Joint Committee on Cancer [26]. 'T' described for how far the primary tumor has grown into the wall of the intestine and whether it has grown into nearby tissues; 'N' gives the extent of spread to nearby (regional) lymph nodes; 'M' indicates whether the cancer has spread (metastasized) to other organs of the body. Numbers or letters appear after T, N and M to provide more details about each of these factors. The numbers 0 through 4 indicate increasing severity. More than 99% over the TNM classifications were clinical rather than pathological but no data were available on the diagnostic criteria used. We limited the present study to cover two of six Swedish healthcare regions (Stockholm-Gotland and Linköping), because of the lowest level of missing data on TNM classes (10%). Once a person's T, N and M categories were determined, the information was combined to 'stage' [26], ranging from stage I (the least advanced) to stage IV (the most advanced). The definitions of stage were as follows: stage I (T1 or T2 N0 M0), stage II (T3 or T4 N0 M0), stage III (any T N1 or N2 M0) and stage IV (any T or N M1).

We analyzed the survival of 10,174 patients diagnosed with colorectal adenocarcinoma in Stockholm-Gotland and Linköping regions from 2000 to 2008, based on the Cox model using time since diagnosis (in months) as the underlying time scale (PROC PHREG, SAS Version 9.2). We investigated the association between CRC-specific mortality and age at diagnosis, gender, socioeconomic status, medical region at diagnosis, location of the tumor, first-degree family history (parent and sibling) of CRC. The data on socioeconomic status was obtained from the national census of 1990 and grouped in six classes: agriculture, worker, blue collar, professional, private and other. The results were summarized as hazard ratios (HRs). Note that the HR compares the death rate of CRC patients to that of the population considering the above variables, including age. Follow-up started at the time of diagnosis. Censoring events were defined as death from other cause than colorectal cancer, diagnosis of second cancer, emigration, and end of the study (December 31, 2008). A p value of <0.05 was considered statistically significant.

The reporting of TNM data to Cancer Registry was lowest in the first years starting in 2002; thus the analysis of TNM data was started from year 2004 and extended through 2008. Ordinal logistic regression models were first used to estimate the risk to be at an advanced stage (stage III/IV vs stage I/II) and then to calculate risks for T (T3/T4 vs T1/T2), N (N1/N2 vs N0) and M (M1 vs M0). For each variable, risks were calculated against the reference category, set at 1.00. Independent predictors were side, age, gender, socioeconomic status, medical region, and first-degree family history of CRC. Analyses were performed using the SAS 9.2 statistical package using the Logistic procedure [27].

## Results

A survival study on 10,174 CRC patients diagnosed from 2000 to 2008 covered the most recent survival trends for Sweden. The results in Table 1 showed that patients diagnosed at younger age survived systematically better, HR being 0.55 for those diagnosed below age 50 years compared to those diagnosed at age 80+ years. Women survived somewhat better than men, HR 0.88. Family history showed no significant effect. Tumor localization had a small but significant effect, transverse colon with the highest risk (1.25) and rectum (1.00) and sigmoid (1.03) the lowest risks. The data were additionally adjusted for socioeconomic status and medical region (data not shown).

**Table 1:** Hazard ratios and 95% confidence intervals (CIs) for cause-specific survival in left- and right-sided colon and rectal adenocarcinoma patients diagnosed from 2000 to 2008

	Number of cases	Number of events	Global P-value	Adjusted HR	95%CI
**Age at diagnosis**			**<.0001**		
<50	566	132		**0.55**	0.46-0.67
50-59	1260	336		**0.69**	0.61-0.79
60-69	2563	592		**0.63**	0.57-0.71
70-79	3040	909		**0.86**	0.78-0.94
80+	2745	889		1.00	
**Gender**			**<0.01**		
Female	4949	1383		**0.88**	0.82-0.95
Male	5225	1475		1.00	
**Family history**					
Yes	534	120		0.89	0.74-1.08
No	9640	2738		1.00	
**Side**			**<0.01**		
Right-side	3735	1122		**1.16**	1.07-1.27
Ascending	2495	733		**1.12**	1.02-1.24
Transverse	1240	389		**1.25**	1.11-1.40
Left-side	2618	707		1.04	0.95-1.15
Descending	281	77		1.15	0.91-1.45
Sigmoid	2337	630		1.03	0.94-1.14
Rectum	3821	1029		1.00	

HRs additionally adjusted for socioeconomic status and medical region.

The significant P values and HRs are shown in bold.

The study population of the TNM study comprised of 6,105 CRC patients is described in Table 2 by the key variables. Young patients (diagnosis before age 50 years) accounted for 4 to 7% of all patients. Patients diagnosed at age 80+ years accounted for 35.4% of the cases in ascending colon and the proportion decreased progressively along colon, reaching 20.6% in rectum. Women accounted for the majority of right-sided cases (55.0%) but not of the left-sided (47.9%) or rectal cancer samples (43.6%). Less than 7% of the patients had a family history of CRC, as diagnosed between 2004 and 2008.

**Table 2:** Characteristics of the study populations diagnosed from 2004 to 2008

	Right Side		Ascending		Transverse		Left Side		Descending		Sigmoid		Rectum	
							
	N	%	N	%	N	%	N	%	N	%	N	%	N	%
**Age at diagnosis**														
<50	102	4.6	63	4.2	39	5.2	103	6.4	12	6.9	91	6.4	137	6.0
50-59	189	8.4	125	8.4	64	8.5	215	13.4	27	15.5	188	13.2	359	15.8
60-69	528	23.6	327	22.0	201	26.8	433	27.1	42	24.1	391	27.4	703	31.0
70-79	644	28.8	445	29.9	199	26.5	485	30.3	54	31.0	431	30.2	602	26.6
80+	775	34.6	527	35.4	248	33.0	364	22.8	39	22.4	325	22.8	466	20.6
Total	2238	100.0	1487	100.0	751	100.0	1600	100.0	174	100.0	1426	100.0	2267	100.0
**Gender**														
Female	1232	55.0	850	57.2	382	50.9	767	47.9	86	49.4	681	47.8	989	43.6
Male	1006	45.0	637	42.8	369	49.1	833	52.1	88	50.6	745	52.2	1278	56.4
Total	2238	100.0	1487	100.0	751	100.0	1600	100.0	174	100.0	1426	100.0	2267	100.0
**Family history**														
Yes	129	5.8	79	5.3	50	6.7	90	5.6	12	6.9	1348	5.5	133	5.9
No	2109	94.2	1408	94.7	701	93.3	1510	94.4	162	93.1	78	94.5	2134	94.1
Total	2238	100.0	1487	100.0	751	100.0	1600	100.0	174	100.0	1426	100.0	2267	100.0

Table 3 shows the distribution of tumor characteristics in CRC patients based on the ordinal logistic regression model. The patients diagnosed before age 50 years showed more advanced stage compared to 80+ year old (OR 1.80) which was explained by more lymph node metastasis (N, OR 1.93) and distant metastasis (M, OR 1.88); all these were significant at p < 0.0001 to p < 0.01, and they showed a systematic age gradient. Right-sided tumors were more advanced than left-sided tumors for stage (1.45 vs. 1.19, rectum is reference, 1.00), T (3.30 vs. 2.12) and N (1.60 vs. 1.16) but not for M. All colonic subsites showed more advanced characteristics compared to the rectum. No sex differences were seen; nor were data on family history significant (not shown).

**Table 3:** Estimated odds ratios (OR) and 95% confidence intervals (CIs) on stage, T, N, M in left- and right-sided colon and rectal adenocarcinoma patients

	STAGE		T		N		M	
	**P**	**OR (95% CI)**	**P**	**OR (95% CI)**	**P**	**OR (95% CI)**	**P**	**OR (95% CI)**
**Age at diagnosis**	**<.0001**		**0.03**		**<.0001**		**<.0.01**	
<50		**1.80 **(1.41-2.29)		1.15 (0.91-1.45)		**1.93 **(1.54-2.42)		**1.88 **(1.40-2.52)
50-59		**1.50 **(1.24-1.81)		**1.27 **(1.07-1.50)		**1.65 **(1.38-1.97)		**1.33 **(1.05-1.68)
60-69		**1.30 **(1.11-1.51)		**1.22 **(1.06-1.40)		**1.46 **(1.26-1.69)		**1.27 **(1.04-1.54)
70-79		**1.17 **(1.01-1.37)		**1.17 **(1.02-1.33)		1.15 (1.00-1.33)		**1.22 **(1.01-1.48)
80+		1.00		1.00		1.00		1.00
**Gender**	0.39		0.51		0.42		0.19	
Female		0.96 (0.87-1.06)		0.96 (0.86-1.08)		1.01 (0.92-1.13)		0.91 (0.80-1.05)
Male		1.00		1.00		1.00		1.00
**Side**	**<.0001**		**<.0001**		**<.0001**		0.25	
Right-side		**1.45 **(1.28-1.64)		**3.30 **(2.89-3.77)		**1.60 **(1.42-1.80)		1.14 (0.98-1.34)
Ascending		**1.45 **(1.26-1.67)		**3.07 **(2.65-3.56)		**1.72 **(1.51-1.97)		1.13 (0.95-1.35)
Transverse		**1.45 **(1.21-1.72)		**3.84 **(3.15-4.68)		**1.38 **(1.17-1.63)		1.17 (0.94-1.46)
Left-side		**1.19 **(1.03-1.36)		**2.12 **(1.85-2.43)		**1.16 **(1.01-1.32)		1.08 (0.91-1.28)
Descending		**1.56 **(1.13-2.15)		**2.70 **(1.90-3.84)		**1.48 **(1.11-1.99)		0.95 (0.63-1.42)
Sigmoid		1.15 (0.99-1.32)		**2.06 **(1.79-2.37)		**1.12 **(0.98-1.28)		1.10 (0.92-1.31)
Rectum		1.00		1.00		1.00		1.00

ORs additionally adjusted for socioeconomic status, medical region and first-degree family history of CRC.

P refers to the overall P value of the variable in the model and the significant P values and ORs are shown in bold.

## Discussion

We showed first that even for CRC diagnosed since 2000, young age at diagnosis was a favorable prognostic factor; the HR was 0.55 for those diagnosed before age 50 years compared to diagnosis at age 80+. Tumor localization played a minor role in survival. Left-sided tumors showing the lowest HR of 1.04 compared to 1.16 for right-sided tumors (rectum was reference, HR 1.00). Among colonic subsites, tumors in transverse colon were associated with the worst survival (1.25) and sigmoid the best survival (1.03). These small differences are in agreement with US data covering the same period [28]. These data were not adjusted for TNM because the classification system was established first in 2002 in the Swedish Cancer Registry and a lot of data were missing in the first year or two. This was a major limitation of the study. Of course, lacking of data on treatment was another limitation.

We used the TNM classification system to address a number of hypotheses: are tumors more advanced in older patients or in men or in those lacking a family history because all these characteristics have been associated with poor survival, particularly in colon cancer [6,10,11,17-20]. For colon and rectal cancers survival has been better for women than for men [4,10]. Unfortunately, the Swedish Cancer Registry collects no detailed data on which diagnostic tests were being used to establish the TNM class.

The results of the study on CRC adenocarcinoma patients with TNM data were clear in concluding that gender and family history were not determinants of tumor characteristics, although in the survival analysis women showed a significant HR of 0.88. Tumor localization was a strong predictor of high T, N and stage but a weaker predictor of M. Rectal tumors had the most favorable characteristics compared to any colonic subsite. Right-sided tumors were more advanced than left-sided tumors for stage, T and N but not for M. The better survival of patients with left-sided tumors may be explained by tumor characteristic in the colon. The relatively favorable tumor characteristics of rectal cancer would have predicted a survival better than that for colon cancer which was not the case. The possible biological differences between right-sided and left-sided tumors have been involved in attempts to explain the low efficacy of colonoscopy to prevent right-sided cancers [21,22]. Although the present data showed somewhat more advanced tumors in the right side, the differences were not large, and for M class no differences were found. Thus tumor characteristics do not appear as likely explanations to the differential colonoscopy success in prevention right-sided and left-sided tumors.

There was a strong and systematic effect of diagnostic age for the ORs for stage, N and M increased stepwise for diagnostic age below 80+ years, the reference category. The ORs were between 1.80 and 1.93 for those diagnosed below 50 years compared to the 80+ reference group. For T the effect was weak and less systematic. The finding of more aggressive tumors among young patients was opposite to the survival data, which were better in young patients. Previous results on diagnostic age and tumor characteristics have been contradictory but the recent data on colon cancer are in agreement with the present results: younger patients present more aggressive and advanced tumors [11,14-16]. It was interesting that no age effect was noted for T, i.e., invasiveness of the primary tumor to near-by tissue structures did not differ between young and old patients. Yet the tumors of young patients were able to invade lymph nodes and send distant metastases. Thus, the overall conclusion is that the poorer survival of old patients in colon cancer is unlikely to be related to delayed diagnosis; delayed diagnosis or presentation may rather be associated with younger patients. It is possible that more aggressive treatment of the young patients plays a role in their better survival [7,8,12,13,29,30]. Yet another puzzling possibility could be that the metastatic tumor cells of young patients may be more sensitive to therapy than those of old patients. However, clinical trials on patients with different ages give no strong support to such a hypothesis [7,8]. The difference in survival could be in response to therapy rather than the therapy itself. Many old patients have co-morbidities that limit the treatment and that increase the risk of death [12,13,29].

## Conclusions

The most recent data from the Swedish Cancer Registry showed systematically more aggressive tumors in young compared to old CRC patients. These results were opposite to the survival data. The poorer survival of old patients in colon cancer was not related to the available tumor characteristics. The more aggressive tumors in the right-sided colon were in partial agreement with the limited colonoscopic success with right-sided tumors.

## Competing interests

The authors declare that they have no competing interests.

## Authors' contributions

KH designed the study and wrote the paper. IS carried out statistical analyses and helped to interpret them. MW helped with statistical analyses. HT helped with statistical analysis. JS provided the data. JLB advised with statistics. All authors read and approved the final manuscript.

## Pre-publication history

The pre-publication history for this paper can be accessed here:

http://www.biomedcentral.com/1471-2407/10/688/prepub
